# Commentary: Experience Sampling Methodology reveals similarities in the experience of passage of time in young and elderly adults

**DOI:** 10.3389/fpsyg.2017.01384

**Published:** 2017-08-10

**Authors:** Adolfo Di Crosta, Pasquale La Malva

**Affiliations:** Department of Psychology, University of Chieti-Pescara Chieti, Italy

**Keywords:** aging, Experience Sampling Methodology, passage of time, time

Few studies have investigated differences in judgments of passage of time between younger and older adults, and the results seem contradictory. Generally, people report that time goes faster as they get older (Lemlich, 1975) which is due to the pressure of perceived time (Janssen et al., 2013). However, Wittmann and Lehnhoff (2005) and Friedman and Janssen (2010) found no clear differences of how passage of time perception changes with aging.

The article by Droit-Volet and Wearden (2015) investigated this issue by using the Experience Sampling Methodology (ESM) technique and combining, for the first time, the assessment of the direct experience of the flow of time in everyday life in both younger and older adults. The study involved 15 younger adults and 14 older adults. Both groups conducted the experiment through the “Experience Sampling Methodology” (ESM) method, which involves submitting a questionnaire to participants at different times of the day using a smartphone provided by the experimenters, and asking them questions regarding the passage of time at different periods of the day. Although this method has been used in other studies regarding the evaluation of perception of time (Conti, 2001; Larson and von Eye, 2006), and it is frequently used in health psychology (e.g., Schwartz and Stone, 1998; Myin-Germeys et al., 2009), the use of it in cognitive psychology is unusual. Participants were also asked questions about the passage of time over longer periods of time (this week, this month, or this year), if they felt that time passed more quickly now than when they were younger or if they felt that time passes more quickly as people get older. The data obtained were analyzed in relation to additional variables, including: the affective state of the participants (positive vs. negative), the level of arousal, the relaxation, the difficulty of the activity, and the degree of attention invested in the activities. Although the passage of time in every-day life was significantly related to affective states and the degree of attention invested in their activities, the results showed no significant difference between the two groups regarding the answers to questions about the passage of time. Interestingly, both groups felt that time passes more quickly as they get older and that time passes faster in the present than when they were younger. Further investigation and different modalities of data collection will be needed in the future to determine whether or not more complex factors, such as the “reference effect,” played a crucial role. In fact, the lack of differences between older and younger adults may have been influenced by the tendency of individuals to make judgments in relation to a salient comparison group rather than in absolute terms when responding to the self-report and peer-report questionnaires (e.g., Youyou et al., 2017). Both, younger and older adults may have probably compared, consciously or unconsciously, the passage of the last day/week/month/year based on their own criteria (i.e., their own recent experience of passage of time which varied between the two groups).

Compared to previous ones, this study emphasizes the innovative use of the ESM method. This method, unlike the paper and pencil questionnaires, offers the possibility of obtaining ecologically large data sets infiltrating in the participants' everyday life to determine directly how they perceive the passage of time (Mehl and Conner, 2012). In the article by Droit-Volet and Wearden there is no measure of the direct experience that participants have on the passage of time on specific daily life activities, but rather the general impression of how time seems to flow in everyday life. In future investigations, this interesting data regarding the impression could be accompanied by an objective and real measurement of the time spent in a certain activity carried out by the participants, to evaluate not only how time has passed (e.g., slow vs. fast) but also how much time has actually passed (e.g., in s, min, h). In particular, the activity carried out by the participant at the time of evaluation could play a very significant role not only in terms of the difficulty of the activity itself, or in the degree of attention invested in it, but also in terms of its affective value (Carstensen et al., 1999; Innamorati et al., 2013; Di Domenico et al., 2015, 2016; Zebrowitz et al., 2015). Based on the extensive literature on emotions and aging (for a review see Fairfield et al., 2015b; Mammarella et al., 2016a), affective contents may affect the performance of participants in different domains including memory, language, and perception (Fairfield et al., 2015a; Altamura et al., 2016; Mammarella et al., 2016b, 2017; Palumbo et al., 2017a,b). In this regard, the study of Droit-Volet et al. consider the overall emotional state of the participant in general (but this may not be determined by the type of activity carried out at that time, but by several other factors). This is related to the fact that, as indicated by the authors, it was not possible to systematically check all the activities carried out by the participants on a daily basis, because they were too varied, but perhaps it would have been advisable to control the inherent value in these activities (Carstensen, 2006) asking the participants to rate the valence of their activities on a scale ranging from very negative to very positive. This would have allowed to divide the activities in positive, negative, and neutral to investigate not only the role of the valence in the perception of the passage of time but also the interaction between affective state and the valence of the activity on experience of passage of time. It is important to remark that in the study of Droit-Volet et al. the authors only measured the affective state of the participants and, although the affective state may play an important role in the perception of passage of time, it is not possible to exclude its independence from the valence of the actions carried out. It is possible, in fact, that a subject with a certain affective state may report that time goes faster when performs positive actions compared to negative ones or vice versa.

In summary, the study of Droit-Volet et al. Emphasizes the innovative use of the ESM method in dealing with issues of cognitive psychology ecologically by taking into account a controversial issue regarding the possible change in the age-related perception of time.

In future research, it may be interesting to investigate, through the use of ecological studies or with more simple and objective paradigm, the role played by emotional factors and therefore not only the general emotional state of the participants but also the value of the activities carried out. More objective studies may include the use of the Mismatch Negativity paradigm (e.g., Chen et al., 2010) to investigate differences between younger and older adults in the perception of time using, for example, neutral, positive, or negative auditory and visual stimuli.

## Author contributions

All authors listed have made a substantial, direct and intellectual contribution to the work, and approved it for publication.

### Conflict of interest statement

The authors declare that the research was conducted in the absence of any commercial or financial relationships that could be construed as a potential conflict of interest.
